# A population-based survey pooled analysis of women engaged in commercial sex in the HIV care cascade in sub-Saharan Africa, 2015–2017

**DOI:** 10.1186/s12982-026-01573-2

**Published:** 2026-02-22

**Authors:** Forrest Larson, Gordon Le, Italia V. Rolle, Madeline Sands, Faith Ussery, Abraham Ater, Abu Abdul-Quader, Gary Sardon, Neena M. Philip, Felix Ndagije, Gerald Mwima, George Mgomella, Fred M. Asiimwe, Tendayi Mharadze, Nzali G. Kancheya, Fatima Glyn Zulu, Mandzisi Mkhontfo

**Affiliations:** 1 Centers for Disease Control and Prevention, National Center for Injury and Prevention and Control, GA Atlanta 30341, USA; 2 Centers for Disease Control and Prevention, Division of Global HIV & TB, Global Health Center, Atlanta, GA, USA; 3 Department of Family Medicine, Oregon Health and Sciences University, Portland, OR, USA; 4 ICAP at Columbia University, New York, NY, USA; 5 Division of Global HIV & TB, Centers for Disease Control and Prevention, Dar es Salaam, Tanzania; 6 Division of Global HIV & TB, Centers for Disease Control and Prevention, Maseru, Lesotho; 7 Division of Global HIV & TB, Centers for Disease Control and Prevention, Harare, Zimbabwe; 8 Division of Global HIV & TB, Centers for Disease Control and Prevention, Lusaka, Zambia; 9 Division of Global HIV & TB, Centers for Disease Control and Prevention, Lilongwe, Malawi; 10 Division of Global HIV & TB, Centers for Disease Control and Prevention, Mbabane, Eswatini

**Keywords:** HIV testing, Africa, Retention in care, Commercial sex, Treatment cascade, Population-based surveys

## Abstract

**Introduction:**

Women who engage in commercial sex (WCS) have a higher prevalence of HIV in sub-Saharan Africa than other women of reproductive age. We aimed to describe the burden of HIV, testing, treatment, and viral load suppression (VLS) coverage among WCS and the correlates of not being engaged in each step of the HIV cascade across six countries between 2015 and 2017.

**Methods:**

Using pooled data from six Population-based HIV Impact Assessment (PHIA) surveys from Eswatini, Lesotho, Malawi, Tanzania, Zambia, and Zimbabwe, sociodemographic, behavioral, and health-related indicators were assessed by commercial sex engagement and steps in the HIV cascade, using prevalence ratios with 95% confidence intervals and statistical significance of *p* < 0.05. All analyses were weighted, and variance was estimated using Taylor series.

**Results:**

Among women aged 18 and older, 3.3% reported selling sex ever or in the last 12 months. HIV prevalence among WCS was 18.3%. HIV status awareness was 65.9% and among those aware of their status, only 57.0% were on treatment. Only half (51.9%) were virally suppressed. Women unmarried and with no reported history of pregnancy who engaged in commercial sex were less likely to be engaged in the HIV care cascade.

**Discussion:**

Overall, women engaged in commercial sex have higher rates of non-engagement at each step in the HIV care cascade and have a higher prevalence of HIV than women in the general population in the countries surveyed. More efforts to engage young, unmarried women engaged in commercial sex who have never had a pregnancy in HIV testing and treatment are needed.

## 1 Introduction

In 2014, the Joint United Nations Program on HIV/AIDS (UNAIDS) set the 90-90-90 targets for 2020 [1], now increased to 95-95-95 by the year 2025, with the goal being that 95% of people living with the human immunodeficiency virus (HIV) will know their HIV status; 95% of all people diagnosed with HIV will be receiving antiretroviral treatment (ART); and 95% of all people on ART will be virally suppressed [2]. These targets were contingent on engagement in the cascade of care, which includes access to testing, timely diagnosis, access to and initiation of treatment, adherence to treatment, and retention in care [2].

There has been extraordinary progress in expanding HIV prevention services and HIV treatment coverage. However, people living with HIV in eastern and southern Africa still make up 54% of all people living with HIV in the world [3]; in 2021, it was estimated that women and adolescent girls made up 63% of eastern and southern Africa’s HIV incidence [4]. Per the UNAIDS definition, people at high risk for HIV (PHR) include men who have sex with men, people who engage in commercial sex and their clients, people who inject drugs, and people in prisons and other closed settings [5]. Across Africa, HIV incidence and prevalence continues to be high among these groups [6]. Despite representing relatively small proportions of the overall population, people at high risk for HIV account for approximately 25% of new infections in east and southern Africa [6, 7]. Between 2016 and 2017, biobehavioral surveys conducted in border areas showed HIV prevalence ranged from 2% to 22% in East Africa for women engaging in commercial sex [8].

Results from attributable fraction analyses demonstrate that women who engage in commercial sex may contribute to ongoing transmission of HIV, impacting overall epidemic trajectories in sub-Saharan Africa [9]. As compared to all women aged 15–49 years, women engaged in commercial sex are 21 times more likely to be infected with HIV globally [3]. Punitive environments with high amounts of violence, criminalization, stigma, discrimination, and social and legal obstacles have been shown to limit and even exclude access to services for HIV prevention, care, and treatment for women engaged in commercial sex [4]. Environments with power imbalances and difficult economic circumstances can result in women engaging in commercial sex having unprotected sex with sexual partners, an inability to negotiate safer sex practices with sexual partners, high prevalence of sexually transmitted infections (STIs) and increased risk of HIV infection, and transmission of HIV to men [10].

An older systematic review with studies from 11 years ago globally estimated that 38% of women living with HIV and engaged in commercial sex were on antiretrovirals and 57% were virally suppressed [11]. While this review was able to calculate ART and viral load suppression (VLS) estimates, the authors also revealed a significant lack of published data on the HIV care cascade among WCS, a need to better characterize the HIV care cascade, and a need to identify both individual and structural barriers encountered in accessing HIV care among WCS [11]. In some settings, WCS may be unaware of their HIV status or if they are aware, they may not be on treatment or be adherent to treatment and thus not virally suppressed [12, 13]. The 2024 global AIDS report suggests that even when the UNAIDS targets are reached through universal test-and-treat interventions at the population level, elevated country and regional HIV incidence may persist in subpopulations [14]. Despite improvements in data collection and survey methodology for representation of people at high risk for HIV within research, a focus on understanding impediments to access of HIV services is limited [6]. An overview of systematic reviews on interventions for increased engagement in the HIV care cascade noted a dearth of research on vulnerable populations and found that the most commonly reported gaps in interventions included insufficient attention to populations most in need of intervention, insufficient primary study robustness, innovation, and quality, and limitations in intervention design including attention to the entirety of the HIV care cascade [15]. For countries to meet the UNAIDS global HIV targets, it is crucial that the HIV cascade be viewed as a continuum, a framework modelling the stages of HIV care through a cascade with five steps; diagnosis, linkage to care, retention in care, adherence to antiretroviral therapy (ART), and viral suppression [16], in both practice and research so that interventions that strengthen the entire cascade can be scaled up.

Bringing women engaged in commercial sex into the HIV care cascade can provide individual health benefits for them as well as reduce HIV incidence in HIV epidemics where the exchange of sex for money or goods contributes to HIV transmission and increases the burden of HIV infection [17]. By taking the factors of non-engagement into account when designing interventions, regimens, and providing health services, there is significant potential to decrease transmission from women engaged in commercial sex to their sexual partners. We utilized the Population-Based HIV Impact Assessments (PHIAs) [18] surveys, conducted in countries with generalized HIV epidemics [19], to identify drivers behind non-engagement at testing, treatment, and VLS among women engaged in commercial sex.

## 2 Methods

### 2.1 Study design and population

PHIA surveys are nationally-representative, cross-sectional household surveys designed to measure adult national HIV incidence and subnational VLS [18, 20, 21]. Survey design, methodology, and weighting procedures are described elsewhere [22–27]. Between 2015 and 2017, the surveys enrolled individuals from all age groups, with the upper age limit varying between countries (from 59 to ≥ 65 years). Data were collected on individuals’ social, behavioral, and demographic characteristics; household members and characteristics; and HIV-related risk factors [28]. Household-based HIV rapid testing was performed according to each country’s national HIV testing algorithm, and results were returned to participants. Those who tested positive were referred to care and treatment.

**Fig. 1:**
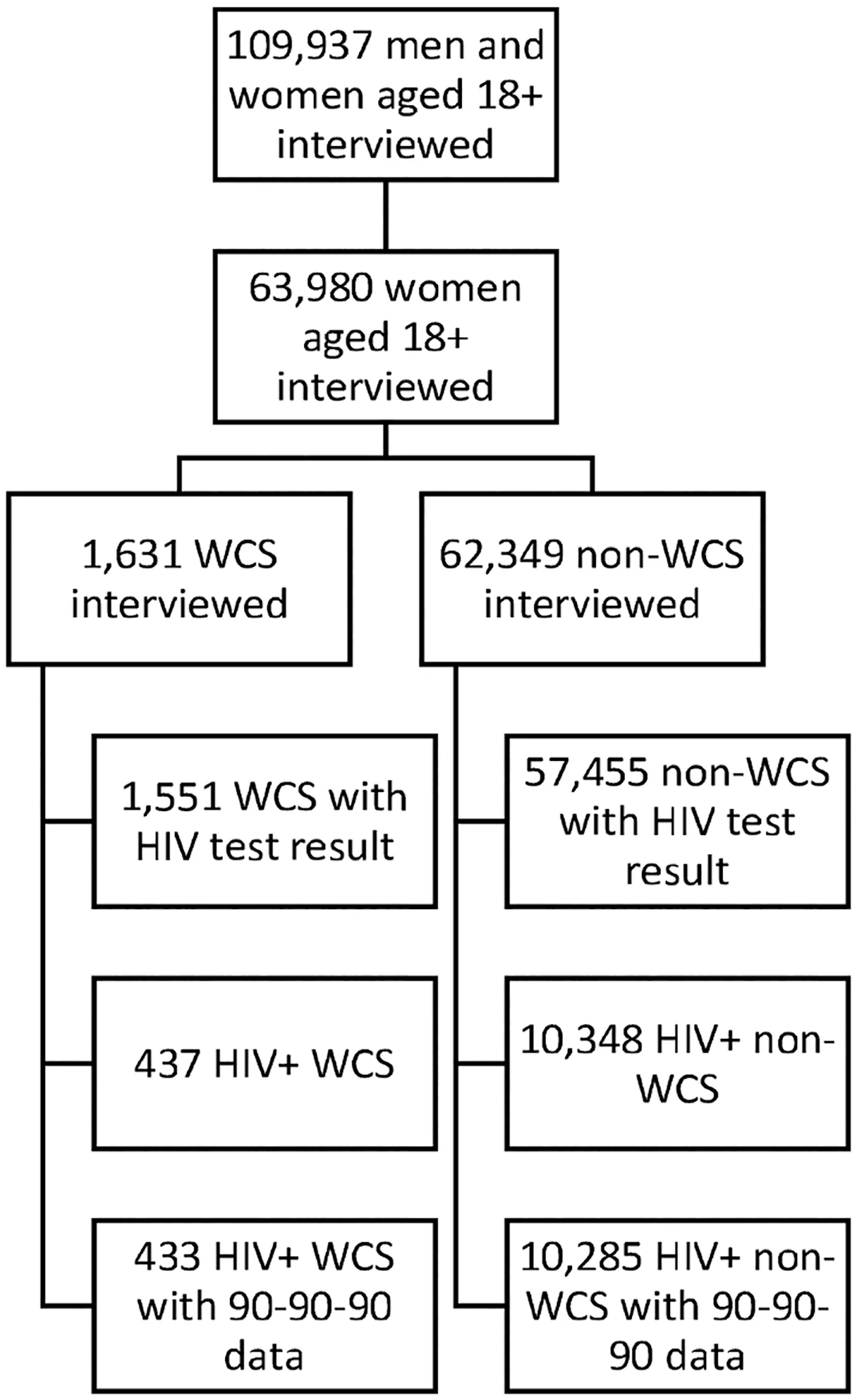
Survey analysis population among women engaging in commercial sex (WCS) and not engaging in commercial sex (non-WCS) who had an HIV test and were included in the self-reported 90-90-90 cascade adjusted with biomarker data

We used PHIA survey data for a multi-country analysis across 6 countries: Eswatini (2016–2017), Lesotho (2016–2017), Malawi (2015–2016), Tanzania (2016–2017), Zambia (2016), and Zimbabwe (2015–2016). Overall, there were 109,937 men and women aged 18 years and older who participated in the surveys. The analysis population is women aged 18 years or older whose survey data included a response on their engagement in commercial sex and had a valid HIV blood test result (Fig. 1).

### 2.2 Definitions

We identified women who engaged in commercial sex based on responses to two questions: “Have you ever sold sex for money?” or “In the last 12 months, have you had/sold sex for money and/or gifts or received payment for sex?“. The question about ever having sold sex was included in only four of the six countries, excluding Tanzania and Zimbabwe (Supplementary Table 1). Women were categorized as having commercial sex engagement (WCS) if they responded “yes” to either or both questions. Women who responded “no” to the question about ever selling sex were categorized as “non-WCS,” indicating they had never engaged in commercial sex. Only condom use among women who engaged in commercial sex during the last instance of sex for money or gifts is reported.

The HIV care cascade (90-90-90 targets) were defined as the proportion aware of HIV serostatus, proportion receiving ART among those aware of serostatus, and proportion virally suppressed among those aware of serostatus and on ART. Women were considered aware of their serostatus if they reported being aware and/or antiretrovirals (ARVs) were detectable. Women were considered on antiretroviral therapy (ART) if they self-reported on ART and/or ARVs were detectable. Women were considered virally suppressed if their viral load was less than 1000 copies/mL. Due to missing data, 67 women (WCS *n* = 4; *n* = 63 non-WCS) living with HIV were excluded from the cascade analyses. The overall viral load suppression was estimated among all women living with HIV, regardless of their knowledge of HIV or use of ART.

We selected variables for analysis based on existing literature, including: age, education, marital status, residence, age at first sex, number of sexual partners in the last 12 months, number of pregnancies, frequency of alcohol consumption, food insecurity, decision maker for health care, and decision maker for monetary spending [29–38]. Further variable selection information can be found in Supplementary Table 2.

### 2.3 Statistical analysis

Data were pooled across the six countries. To account for the complex survey design, all estimates were weighted, and variances were estimated using the Taylor series linearization method. Descriptive statistics were analyzed using SAS (version 9.4, SAS Institute, Cary, NC). R version 4.1.1, using the survey package, was used to fit log-linear regression models to estimate the prevalence ratios (PRs) and 95% confidence intervals (CI). To determine the impact of engagement in commercial sex on cascade outcomes (serostatus awareness, ART status, viral load suppression), we first fit unadjusted bivariate models for each outcome. Reference groups were chosen based on the literature [39, 40]. A selection cut-off of p-value < 0.1 in each of the bi-variate analysis was used to determine the variable selection for the final multivariate model by engagement in commercial sex. The final multivariate model included age as a confounder based on the literature [32] and results of a priori variable exploration. Many variables with selection cut-off of *p* < 0.1 did not have a large enough sample size across response groups or were not comparable across all PHIA countries for a multivariate model, and thus were not included in the final adjusted model, although similar studies might have included them [29, 33]. Prevalence ratios were considered significant at p-value<0.05.

### 2.4 Ethical approval

The surveys were conducted in accordance to the Declaration of Helsinki. Each survey was reviewed and approved by in-country ethics and regulatory bodies and the institutional review boards (IRBs) of Columbia University, Westat, and the U.S. Centers for Disease Control and Prevention (CDC)^1^. All participants provided written informed consent before participating in the surveys.

## 3 Results

### 3.1 Population characteristics

Among 63,980 women aged 18 years and older who were interviewed and had a valid HIV test, almost half (43.3% [95% CI: 42.7–43.9]) were less than 30 years of age, 68.1% (95% CI: 67.2–69.0) had no education or only primary education, and 63.3% (95% CI: 62.4–64.1) were either married or living together with partner (Table 1). Two-thirds (62.5% [95% CI: 60.3–64.7]) resided in a rural area and 88.4% (95% CI: 87.9–88.8) reported one or more pregnancies previously. Overall, 3.3% (95% CI: 3.0–3.6) of women reported ever selling sex or selling sex in the last 12 months (WCS) while 96.7% (95% CI: 96.4–97.0) did not (non-WCS).

### 3.2 Characteristics of women living with HIV

HIV prevalence among women engaged in commercial sex was 18.2% (95% CI: 15.7–20.8) and 11.3% of women did not engage’ in commercial sex (95% CI: 10.9–11.7). Characteristics of women living with HIV, categorized by commercial sex engagement, are shown in Table 2. One-third (33.3% [95% CI: 25.9–40.6]) of WCS living with HIV were aged less than 30 years, compared to 24.1% (95% CI: 22.9–25.3) of non-WCS living with HIV. Approximately three-quarters of WCS (76.9% [95% CI: 71.6–82.2]) had either no education or primary education, compared to 64.6% of non-WCS (95% CI: 63.1–66.1). A third (30.6% [95% CI: 22.6–38.5]) of WCS had experienced food insecurity in the last 4 weeks compared to one fifth (22.8%) of non-WCS (95% CI: 20.9–24.8). One-third (33.4% [95% CI: 27.2–39.6]) of WCS reported first having sex at 15 years of age or younger, with 47.0% (95% CI: 38.4–55.7) reporting two or more partners in the last year. Comparatively, 21.5% (95% CI: 20.3–22.7) of non-WCS reported first having sex at 15 years of age or younger, and 6.4% (95% CI: 5.6–7.2) reported two or more partners in the last year. Almost half of WCS (47.2% [95% CI: 38.4–56.1]) reported not using condoms the last time they had sex for money or gifts.

### 3.3 HIV prevalence, viral load suppression, and HIV care cascade

Among women who reported engaging in commercial sex, 18.2% (95% CI: 15.7–20.8) were living with HIV whereas 11.3% (95% CI: 10.9–11.7) of women who did not report engaging in commercial sex were living with HIV (Table 3). Overall, VLS prevalence was lower among WCS at 55.4% (95% CI: 47.0–63.7%) compared to non-WCS at 63.9% (95% CI: 62.6%−65.3%).

**Fig. 2:**
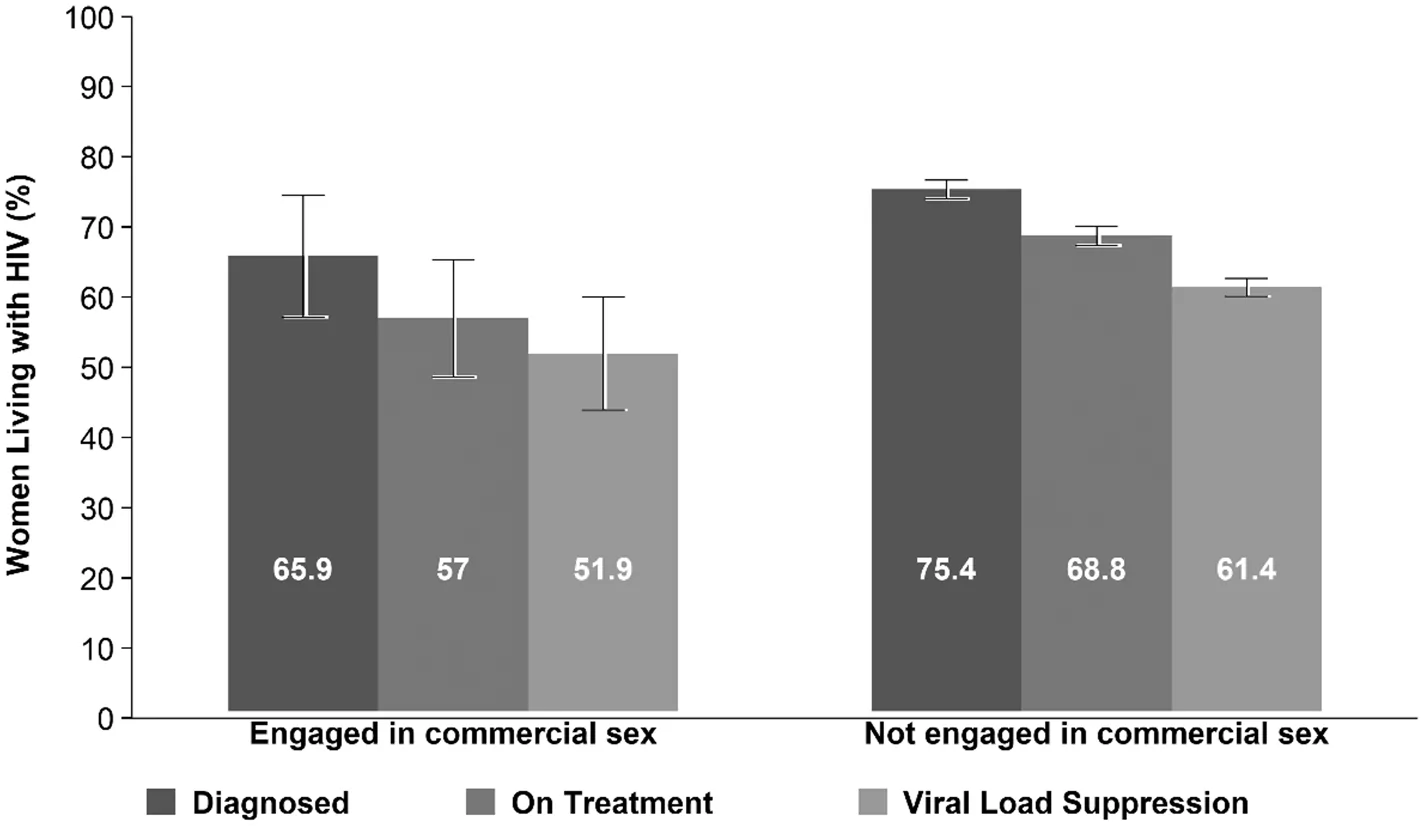
90-90-90 targets among women aged 18 years and older living with HIV based on their self-reported HIV status and ART use, adjusted by detectable ARVs in blood, by commercial sex engagement, PHIA Surveys, 2015–2017

Of those aware of their HIV status, 65.9% (95% CI: 57.2–74.5) of WCS were aware of their HIV status compared to 75.4% (95% CI: 74.0–76.7) of non-WCS (Fig. 2). Among those aware of their HIV status, only 57.0% (95% CI: 48.6–65.3) of WCS were on ART compared to 68.8% (95% CI: 67.4–70.1) of non-WCS. Among those aware of their HIV status and on treatment, only half (51.9%; 95% CI: 43.9–60.0) were virally suppressed compared to 61.4% (95% CI: 60.1–62.7) of non-WCS.

#### 3.3.1 Correlates associated with non-engagement in HIV care cascade

Crude and adjusted prevalence ratios for non-engagement in the HIV care cascade are presented in Table 4 (WCS) and Table 5 (non-WCS). In the multivariate models for both WCS and non-WCS, two correlates were associated with increased likelihood of cascade non-engagement: marital status and number of pregnancies. Among never married WCS and non-WCS, there was an increased likelihood of having unsuppressed viral load compared to WCS and non-WCS who were married or living with their partner. Among WCS, the increase in likelihood was 37% (95% CI: 0.97–1.94), and among non-WCS the increase in likelihood was 17% (95% CI: 1.06–1.28). When women reported no pregnancies, the likelihood of being unaware of their HIV status was higher, 83% among WCS (95% CI: 1.13–2.95) and 67% among non-WCS (95% CI: 1.44–1.94). WCS with no reported pregnancies were 50% (aPR: 1.50 [95% CI: 1.01–2.23]) more likely to be not on ART when compared to WCS who reported at least 1 pregnancy. This finding is similar to the 46% (95% CI: 1.29–1.66) increased likelihood of not being on ART observed in non-WCS. Additionally, WCS (aPR: 1.46 [95% CI: 1.05–2.05]) and non-WCS (aPR: 1.36 [95% CI: 1.23–1.51]) with no previous pregnancies were found to be more likely to have an unsuppressed viral load compared to those who had previous history of pregnancy.

## 4 Discussion

It is well known that women engaged in commercial sex are at a greater risk of non-engagement from the HIV care cascade [41]. HIV care cascade non-engagement and HIV prevalence among this pooled analysis demonstrates health differences between women engaged in commercial sex and general women populations, aligning with existing data from the countries of interest [34, 41, 42]. Gaps in progress toward the UNAIDS targets are particularly evident when it comes to serostatus awareness and ART uptake with non-engagement significantly higher in populations reporting engagement in commercial sex. Peer support could lead to better serostatus awareness and ART uptake among those engaged in commercial sex [43] and discrimination by health care workers may create further differences between women engaged in commercial sex and women not engaged in commercial sex with ART uptake [44]. Those engaged in commercial sex have a higher percentage of unsuppressed viral load when no correlates are considered, emphasizing a need for targeted approaches to increase serostatus awareness and ART uptake for VLS [43].

Never married women and women having no past or current pregnancies were found to be significantly more likely to be non-engaged in the HIV care cascade at all points. The literature suggests a correlation between age and retention in care [45], potentially due to structural and age-related legal barriers among adolescent women such as age of consent laws [4], inadequate participation of young women in the development of community-led programs, or insufficient peer-led outreach and digital technology approaches to ensure adolescent girls and young women are reached with effective services early [46]. Further, antenatal care surveillance across sub–Saharan Africa shows younger and unmarried women are less likely to become pregnant and thus less likely to start engagement in the cascade through antenatal care access points [47, 48]. Pregnant women are more likely to be identified and linked to care for prevention of mother-to-child transmission of HIV, and generally, HIV services are integrated into antenatal care. The DREAMS (Determined, Resilient, Empowered, AIDS-free, Mentored and Safe) partnership is a service that aims to meet the needs of adolescent girls and young women and is implemented within the six countries in this analysis [49]. DREAMS outcomes have frequently been measured through HIV diagnoses in antenatal care (ANC), but sampling adolescent girls and young women from ANC excludes individuals who are not receiving services from ANC [50, 51]. In this analysis, young women participating in DREAMS could not be identified. Outreach may not have been comprehensive enough to reach young, unmarried women with no history of pregnancy [52, 53]. Our results might indicate a lack of women’s health services unaffiliated with pregnancy and suggest a need for subsidized or free women’s wellness checks throughout the lifespan [46]. Emphasizing this, the UNAIDS Global AIDS Strategy for 2021–2026 calls for expansion of comprehensive programs to address gaps in HIV care for women engaged in commercial sex, including increased sexual and reproductive health services, especially the provision of alternative programs using creative approaches to reach and engage women engaged in commercial sex in HIV, sexual, and reproductive health and related prevention initiatives and services [46].

It is clear that women who engage in commercial sex are an important population that need tailored HIV interventions; however, there are often insufficient data or no data on the size of these populations [7]. Population-based surveys, especially data on the HIV care cascade, lack a focus on groups such as women engaged in commercial sex, and challenges exist with the collection of program-based data, as individuals relocate between health centers and/or experience discomfort disclosing engagement in commercial sex to program staff [54]. Additionally, biobehavioral surveys may still be an underestimate of women engaged in commercial sex population size because they are hard to reach and the areas where the surveys are conducted may not be nationally representative [55]. Research suggests that more data are needed on WCS populations, especially those from non-research settings, and there are substantial gaps in the spatial distribution of cascade estimates among WCS [11]. Even though the data reported are not recent, we used general population-based surveys to identify this population of women to fill in the gaps that localized biobehavioral surveys are not able to capture for countries where there is a historic lack of data.

There are some limitations of this analysis. PHIA methodology was not designed to estimate the UNAIDS 90-90-90 outcomes among women living with HIV and engaged in commercial sex. Data on violence, stigma, and discrimination are mostly missing for this population, which may be important for the care cascade estimates. Additionally, there was no data on recency or frequency of commercial sex, and as a result, cascade non-engagement may look different than if these data were available for inclusion. Because women engaged in commercial sex are often a hidden population [56], they may be under-sampled since the survey design did not target this population specifically. There is also likely an under-reporting of engagement in commercial sex due to fear of stigma and discrimination [44]. Misclassification of commercial sex engagement among women who have ever sold sex may have occurred, as their past engagement might not reflect their current involvement. Face-to-face interviews collecting data on commercial sex may introduce reporting bias and lead to underestimates of this population. Additionally, it is important to note that, as HIV prevalence and cascade indicators were measured using data from 2015 to 2017, findings of this analysis may not reflect current engagement in the HIV care cascade. Ongoing reassessment of HIV prevalence and cascade indicators using more recent data is warranted. Despite these limitations, using data from PHIAs is an opportunity to gain additional insight into a hidden population. The focus of this analysis aligns with previous directions of the U.S. President’s Emergency Plan for AIDS Relief (PEPFAR) to close the prevention and treatment gap for people living with HIV to achieve UNAIDS 95-95-95 targets by 2030 [57].

## 5 Conclusions

The correlates of HIV care cascade non-engagement among women engaged in commercial sex have important implications, and a clear difference exists between women engaged in commercial sex and the general women population. A consideration of the unique needs for this group when designing HIV testing and care services could help in the engagement of these women from the point of testing to VLS. Exploration of existing differences, especially surrounding provision of services targeting ART adherence for VLS may also increase the impact of HIV adherence programs and help control the spread of HIV, while special consideration of the needs of young, unmarried, women engaged in commercial sex who have never been pregnant, and exploration of barriers to care and potential engagement points outside of ANC could lead to additional engagement in the HIV care cascade among populations that are missed. Greater exploration of the effects of DREAMS or DREAMS-like programs on engagement in the HIV care continuum could help reach young, unmarried women with no history of pregnancy [52, 53]. To achieve the UNAIDS 95–95–95 targets to end AIDS as a public health threat by 2030 in sub-Saharan Africa, a renewed focus on women- based health care throughout the lifespan and regardless of pregnancy status suggests additional consideration.

## Supplementary Material

Supplementary Table 1

Supplementary Table 2

The online version contains supplementary material available at https://doi.org/10.1186/s12982-026-01573-2.

Supplementary Material 1

Supplementary Material 2

## Figures and Tables

**Table 1:** Sociodemographic characteristics of women aged 18 years and older in the Population-based HIV impact assessment surveys (PHIA) conducted in six African countries, 2015–2017

Characteristics	%	95% CI^c^	*N*
*Demographics*			
Age at interview (years)			63,980
18–29	43.3	42.7–43.9	
30+	56.7	56.1–57.3	
Highest level of education completed			63,923
No education or primary	68.1	67.2–69.0	
Secondary or higher	31.9	31.0–32.8	
Marital status			63,837
Never married	15.9	15.3–16.5	
Married, living together	63.3	62.4–64.1	
Widowed, divorced, separated	20.8	20.2–21.4	
Residence			63,980
Urban	37.5	35.3–39.7	
Rural	62.5	60.3–64.7	
*Health-Related Indicators*			
Number of pregnancies			63,638
0 pregnancies	11.6	11.2–12.1	
1 + pregnancies	88.4	87.9–88.8	
Food insecurity in the last 4 weeks			40,513
Yes	18.1	17.0–19.2	
No	81.9	80.8–83.0	
HIV Status			59,006
Positive	11.6	11.2–12.0	
Negative	88.4	88.0–88.8	
*Risk Behaviors*			
Age at first sex (years)			58,967
Less than 15	20.7	20.1–21.3	
16–17	26.0	25.4–26.6	
18 and older	53.2	52.4–54.1	
No. sexual partners in last 12 months			59,369
0–1	94.3	93.9–94.6	
2 or more	5.7	5.4–6.1	
Ever sold sex^c^			29,359
Yes	3.4	3.0–3.7	
No	96.6	96.3–97.0	
Sold sex in the last 12 months			25,353
Yes	4.6	4.2–5.1	
No	95.4	94.9–95.8	
Sell sex ever or in the last 12 months			63,980
Yes	3.3	3.0–3.6	
No	96.7	96.4–97.0	
Ever tested for HIV prior to interview			63,841
Yes	81.9	81.4–82.5	
No	18.1	17.5–18.6	
*Decision and Empowerment Norms*			
Health care decision-maker			39,513
Self	37.6	36.7–38.6	
Spouse/Partner/someone else	19.6	18.8–20.3	
Both	42.8	42.0–43.7	
Financial decision-maker			39,506
Self	21.8	21.0–22.6	
Spouse/Partner/someone else	25.5	24.7–26.3	
Both	52.7	51.8–53.6	

HIV, Human Immunodeficiency Virus; CI, confidence interval; PHIA, Population-based HIV Impact Assessment

N is unweighted

Percents are weighted

c Tanzania and Zimbabwe excluded

**Table 2:** Sociodemographic characteristics of women aged 18 years and older living with HIV, by commercial sex engagement, in six African countries, PHIA Surveys, 2015–2017

Characteristics	HIV Positive non-WCS			HIV Positive WCS		
	%	95% CI	*N*	%	95% CI	*N*
*Demographics*						
Age at interview (years)			10,348			437
18–29	24.1	22.9–25.3		33.3	25.9–40.6	
30+	75.9	74.7–77.1		66.7	59.4–74.1	
Highest level of education completed			10,339			437
No education or primary	64.6	63.1–66.1		76.9	71.6–82.2	
Secondary or higher	35.4	33.9–36.9		23.1	17.8–28.4	
Marital status			10,322			433
Never married	10.7	9.9–11.6		9.2	5.3–13.0	
Married, living together	51.7	50.2–53.2		39.7	33.8–45.6	
Widowed, divorced, separated	37.6	36.0–39.1		51.1	44.8–57.5	
Residence			10,348			437
Urban	46.6	44.3–48.9		48.8	39.8–57.9	
Rural	53.4	51.1–55.7		51.2	42.1–60.2	
*Health-Related Indicators*						
Number of pregnancies			10,318			435
0 pregnancies	5.3	4.7–5.9		6.9	2.9–10.9	
1 + pregnancies	94.7	94.1–95.3		93.1	89.1–97.1	
Food insecurity in the last 4 weeks			6,767			276
Yes	22.8	20.9–24.8		30.6	22.6–38.5	
No	77.2	75.2–79.1		69.4	61.5–77.4	
*Risk Behaviors*						
Age at first sex (years)			9,874			436
Less than 15	21.5	20.3–22.7		33.4	27.2–39.6	
16–17	25.7	24.4–27.0		32.1	25.0–39.1	
18 and older	52.8	51.3–54.3		34.5	27.7–41.3	
No. sexual partners in last 12 months			9,981			430
0–1	93.6	92.8–94.4		53.0	44.3–61.6	
2 or more	6.4	5.6–7.2		47.0	38.4–55.7	
Sold sex in the last 12 months						437
Yes	NA	NA	NA	77.5	73.0–82.0	
No	NA	NA	NA	22.5	18.0–27.0	
Condom use last time had sex for money and/or gifts^b^						337
Yes	NA	NA	NA	52.8	43.9–61.6	
No	NA	NA	NA	47.2	38.4–56.1	
Ever tested for HIV prior to interview			10,339			436
Yes	93.5	92.7–94.4		94.1	91.3–96.9	
No	6.5	5.6–7.3		5.9	3.1–8.7	
*Decision and Empowerment Norms*						
Health care decision-maker			5,257			167
Self	38.4	36.4–40.3		53.8	42.7–64.9	
Spouse/Partner/someone else	18.3	16.8–19.8		14.6	7.2–21.9	
Both	43.4	41.3–45.4		31.7	22.0–41.4	
Financial decision-maker			5,258			167
Self	24.0	22.3–25.7		33.8	22.7–44.9	
Spouse/Partner/someone else 23.3	21.5–25.1		14.2	8.1–20.4	
Both	50.6–54.7		52.0	40.5–63.4	

HIV, Human Immunodeficiency Virus; WCS, women engaged in commercial sex; non-WCS, women not engaged in commercial sex; CI, confidence interval

Percents are weighted

N is unweighted

a Tanzania and Zimbabwe excluded

b Only asked in the WCS sample

**Table 3:** HIV prevalence and viral load suppression among women aged 18 years and older living with HIV by commercial sex engagement, PHIA Surveys, 2015–2017

	WCS *(N=437)*		Non-WCS *(N = 10,348)*	
	%	95% CI	%	95% CI
HIV Prevalence	18.2	15.7–20.8	11.3	10.9–11.7
Viral Load Suppression	55.4	47.0–63.7	63.9	62.6–65.3

HIV, Human Immunodeficiency Virus; WCS, women engaged in commercial sex; non-WCS, women not engaged in commercial sex; CI, confidence interval

Percents are weighted

**Table 4:** Correlates of non-engagement in the HIV cascade among women living with HIV who engage in commercial sex, PHIA Surveys, 2015–2017

Characteristics	Unaware of HIV status		Not on ART		Not virally suppressed	
	cPR (95% CI)	aPR (95% CI)	cPR (95% CI)	aPR (95% CI)	cPR (95% CI)	aPR (95% CI)
*Demographics*						
Age at interview (years)						
18–29	1.27 (0.77–2.12)		1.36 (0.93–2.01)		1.29 (0.92–1.79)	
30+	*Ref*		*Ref*		*Ref*	
Highest level of education completed						
No education or primary	1.02 (0.64–1.66)		0.92 (0.65–1.31)		0.92 (0.68–1.25)	
Secondary or higher	*Ref*		*Ref*		*Ref*	
Marital status						
Never married	1.53 (0.92–2.55)	1.47 (0.89–2.43)	1.4 (0.92–2.14)	1.29 (0.84–1.98)	**1.47 (1.04–2.06)**	**1.37 (0.97–1.94)**
Married, living together	*Ref*	*Ref*	*Ref*	*Ref*	*Ref*	*Ref*
Widowed, divorced, separated	0.82 (0.51–1.30)	0.84 (0.53–1.31)	0.96 (0.66–1.34)	1.01 (0.71–1.43)	0.96 (0.69–1.33)	0.99 (0.73–1.36)
Residence						
Urban	*Ref*		*Ref*		*Ref*	
Rural	0.95 (0.56–1.62)		0.79 (0.54–1.15)		0.82 (0.59–1.14)	
*Health-Related Indicators*						
Number of pregnancies						
0 pregnancies	**1.84 (1.11–3.06)**	**1.83 (1.13–2.95)**	1.51 (0.95–2.40)	**1.50 (1.01–2.23)**	**1.47 (0.99–2.17)**	**1.46 (1.05–2.05)**
1 + pregnancies	*Ref*	*Ref*	*Ref*	*Ref*	*Ref*	*Ref*
Food insecurity in the last 4 weeks						
Yes	0.81 (0.51–1.29)	0.84 (0.53–1.31)	0.8 (0.53–1.21)	0.84 (0.58–1.22)	0.84 (0.58–1.21)	0.88 (0.63–1.22)
No	*Ref*	*Ref*	*Ref*	*Ref*	*Ref*	*Ref*
*Risk Behaviors*						
Age at first sex (years)						
Less than 15	0.89 (0.49–1.60)	0.89 (0.49–1.59)	0.91 (0.58–1.42)	0.92 (0.60–1.43)	0.96 (0.66–1.39)	0.96 (0.67–1.39)
16–17	*Ref*	*Ref*	*Ref*	*Ref*	*Ref*	*Ref*
18 and older	1.27 (0.71–2.27)	1.37 (0.79–2.36)	1.09 (0.69–1.73)	1.19 (0.78–1.83)	1.02 (0.67–1.55)	1.09 (0.74–1.62)
No. sexual partners in last 12 months						
0–1	0.83 (0.47–1.47)	0.85 (0.49–1.45)	0.74 (0.46–1.17)	0.7 (0.49–1.14)	0.78 (0.53–1.15)	0.79 (0.55–1.13)
2 or more	*Ref*	*Ref*	*Ref*	*Ref*	*Ref*	*Ref*
Condom use last sex for money and/or gifts						
Yes	*Ref*		*Ref*		*Ref*	
No	1.28 (0.75–2.18)		1.05 (0.68–1.62)		0.95 (0.64–1.41)	
*Decision and Empowerment Norms*						
Health care decision-maker						
Self	*Ref*	*Ref*	*Ref*	*Ref*	*Ref*	*Ref*
Spouse/Partner/someone else	**0.37 (0.14–1.00)**	0.38 (0.14–1.02)	0.58 (0.30–1.13)	0.60 (0.31–1.17)	0.61 (0.35–1.07)	0.63 (0.37–1.10)
Both	**0.29 (0.12–0.71)**	**0.29 (0.12–0.72)**	**0.46 (0.23–0.89)**	**0.46 (0.24–0.91)**	**0.50 (0.28–0.91)**	**0.5 (0.28–0.93)**
Financial decision-maker						
Self	*Ref*	*Ref*	*Ref*	*Ref*	*Ref*	*Ref*
Spouse/Partner/someone else	1.06 (0.43–2.63)	1.07 (0.43–2.62)	1.15 (0.49–2.69)	1.18 (0.52–2.70)	0.96 (0.45–2.02)	0.99 (0.47–2.05)
Both	1.04 (0.45–2.44)	1.07 (0.47–2.45)	1.29 (0.61–2.73)	1.32 (0.63–2.76)	1.1 (0.60–2.05)	1.14 (0.62–2.10)

ART, antiretroviral treatment; cPR, crude prevalence ratios; aPR, adjusted prevalence ratio; CI, confidence interval; Ref, reference

Bold values are statistically significant at p-value < 0.05

**Table 5:** Correlates of non-engagement in the HIV cascade among women living with HIV who do not engage in commercial sex, PHIA Surveys, 2015–2017

Characteristics	Unaware of HIV status		Not on ART		Not virally suppressed	
	cPR (95% CI)	aPR (95% CI)	cPR (95% CI)	aPR (95% CI)	cPR (95% CI)	aPR (95% CI)
*Demographics*						
Age at interview (years)						
18–29	**1.79 (1.61–1.99)**		**1.67 (1.53–1.82)**		**1.58 (1.46–1.70)**	
30+	Ref		Ref		Ref	
Highest level of education completed						
No education or primary	1.07 (0.96–1.20)		0.96 (0.89–1.05)		0.97 (0.90–1.04)	
Secondary or higher	Ref		Ref		Ref	
Marital status						
Never married	**1.37 (1.18–1.59)**	**1.21 (1.04–1.40)**	**1.33 (1.18–1.49)**	**1.19 (1.06–1.34)**	**1.27 (1.15–1.41)**	**1.17 (1.06–1.28)**
Married, living together	Ref	Ref	Ref	Ref	Ref	Ref
Widowed, divorced, separated	0.99 (0.89–1.10)	1.08 (0.97–1.21)	0.98 (0.89–1.07)	1.06 (0.97–1.17)	0.97 (0.89–1.05)	1.04 (0.96–1.14)
Residence						
Urban	Ref		Ref		Ref	
Rural	1.03 (0.92–1.14)		0.99 (0.91–1.08)		0.99 (0.92–1.06)	
*Health-Related Indicators*						
Number of pregnancies						
0 pregnancies	**1.99 (1.71–2.32)**	**1.67 (1.44–1.94)**	**1.71 (1.50–1.95)**	**1.46 (1.29–1.66)**	**1.55 (1.39–1.73)**	**1.36 (1.23–1.51)**
1 + pregnancies	Ref	Ref	Ref	Ref	Ref	Ref
Food insecurity in the last 4 weeks						
Yes	**0.84 (0.72–0.98)**	**0.86 (0.75–0.98)**	**0.85 (0.74–0.97)**	**0.86 (0.75–0.98)**	**0.88 (0.78–0.99)**	**0.89 (0.79–1.00)**
No	Ref	Ref	Ref	Ref	Ref	Ref
*Risk Behaviors*						
Age at first sex (years)						
Less than 15	1.05 (0.90–1.23)	1.08 (0.93–1.25)	1.06 (0.93–1.21)	1.08 (0.96–1.23)	1.07 (0.96–1.20)	1.09 (0.98–1.21)
16–17						
18 and older	0.94 (0.82–1.07)	0.98 (0.86–1.12)	0.95 (0.85–1.06)	0.99 (0.89–1.10)	0.96 (0.87–1.05)	0.99 (0.90–1.09)
No. sexual partners in last 12 months						
0–1	**0.59 (0.50–0.71)**	**0.64 (0.55–0.76**)	**0.64 (0.56–0.73)**	**0.64 (0.55–0.76)**	**0.73 (0.65–0.83)**	**0.78 (0.70–0.88)**
2 or more	Ref	Ref	Ref	Ref	Ref	Ref
*Decision and Empowerment Norms*						
Health care decision-maker						
Self	Ref	Ref	Ref	Ref	Ref	Ref
Spouse/Partner/someone else	0.88 (0.71–1.08)	0.87 (0.71–1.07)	0.91 (0.77–1.08)	0.91 (0.77–1.08)	0.9 (0.79–1.03)	0.91 (0.79–1.03)
Both	**0.80 (0.68–0.94)**	**0.80 (0.68–0.94)**	**0.82 (0.72–0.94)**	**0.82 (0.72–0.94)**	**0.83 (0.74–0.93)**	**0.84 (0.75–0.93)**
Financial decision-maker						
Self	Ref	Ref	Ref	Ref	Ref	Ref
Spouse/Partner/someone else	0.94 (0.75–1.17)	0.87 (0.70–1.09)	0.97 (0.80–1.16)	0.91 (0.76–1.09)	0.97 (0.84–1.11)	0.93 (0.81–1.07)
Both	0.87 (0.72–1.06)	0.85 (0.70–1.02)	0.93 (0.79–1.09)	0.9 (0.77–1.05)	0.88 (0.78–1.01)	**0.87 (0.76–0.98)**

ART, antiretroviral treatment; cPR, crude prevalence ratios; aPR, adjusted prevalence ratio; CI, confidence interval; Ref, reference

Bold values are statistically significant at p-value <. 05

## Data Availability

All data for the surveys used in this analysis can be obtained from ICAP at Columbia University, the PHIA project (https://phia.icap.columbia.edu/).
