# Supplementary material for: A population-based survey pooled analysis of women engaged in commercial sex in the HIV care cascade in sub-Saharan Africa, 2015–2017
Source: Discov Public Health. Author manuscript; Available in PMC 2026 Sep 19. (PMC13588663; doi:10.1186/s12982-026-01573-2)
Supplement: Supplementary file 1 [file NIHMS2157507-supplement-Supplementary_Table_1.docx]

**Supplementary Table 1** Women aged 18 years and older living with HIV by six PHIA countries, a disaggregation of PHIA questions to assess engagement in commercial sex.

|  | **Ever sold sex** | | | | | **Sold sex in the last 12 months** | | | | | | **Sell sex ever and/or sell sex 12 months** | | | | | | **No sex work** | | | | |
| --- | --- | --- | --- | --- | --- | --- | --- | --- | --- | --- | --- | --- | --- | --- | --- | --- | --- | --- | --- | --- | --- | --- |
| **Country** | **n** | **weighted n** | **weighted %** | **95% CI** | | **n** | **weighted n** | **weighted %** | **95% CI** | | **n** | | **weighted n** | **weighted %** | **95% CI** | | **n** | | **weighted n** | **weighted %** | **95% CI** | |
| Eswatini | 8 | 650 | 0.9 | 0.4 | 1.5 | 1 | 83 | 0.07 | 0.0 | 0.2 | 8 | | 650 | 0.4 | 0.1 | 0.66 | 1,988 | | 121,456 | 4.4 | 4.0 | 4.7 |
| Lesotho | 105 | 8835 | 12.4 | 9.7 | 15.1 | 64 | 5,315 | 4.2 | 3.0 | 5.4 | 105 | | 8,835 | 5.3 | 4.0 | 6.6 | 2,035 | | 159,945 | 5.8 | 5.4 | 6.1 |
| Malawi | 115 | 38,157 | 53.7 | 47.2 | 60.2 | 57 | 18,646 | 14.6 | 8.8 | 20.5 | 115 | | 38,157 | 23.1 | 17.2 | 289 | 1,383 | | 461,402 | 16.5 | 15.2 | 17.9 |
| Tanzania^c^ | - | - | - | - | - | 101 | 78,832 | 61.9 | 52.6 | 71.2 | 101 | | 78,832 | 47.6 | 38.4 | 56.9 | 1,150 | | 873,745 | 31.4 | 29.2 | 33.6 |
| Zambia | 64 | 23,463 | 33.0 | 26.6 | 39.4 | 25 | 9,008 | 7.1 | 3.9 | 10.2 | 64 | | 23,463 | 14.2 | 9.9 | 18.4 | 1,597 | | 520,534 | 18.8 | 17.5 | 20.0 |
| Zimbabwe^c^ | - | - | - | - | - | 44 | 15,549 | 12.2 | 7.7 | 16.7 | 44 | | 15,549 | 9.4 | 5.9 | 12.9 | 2,195 | | 638,210 | 23.2 | 21.9 | 24.4 |
| **Total** | 292 | 71,105 | 100 |  |  | 292 | 127,434 | 100 |  |  | 437 | | 165,487 | 100 |  |  | 10,348 | | 2,775,292 | 100 |  |  |

^c^Tanzania and Zimbabwe surveys did not include ever sold sex as a question.

HIV, Human Immunodeficiency Virus; PHIA, Population-based HIV Impact Assessment survey
