# Supplementary material for: A population-based survey pooled analysis of women engaged in commercial sex in the HIV care cascade in sub-Saharan Africa, 2015–2017
Source: Discov Public Health. Author manuscript; Available in PMC 2026 Sep 19. (PMC13588663; doi:10.1186/s12982-026-01573-2)
Supplement: Supplementary file 2 [file NIHMS2157507-supplement-Supplementary_Table_2.docx]

**Supplementary Table 2** Explanation of correlates used within analysis and variable chosen from Population-based HIV Impact Assessment surveys to use as correlate.

| **Correlate** | **Variable(s) used** | **Notes on correlate** |
| --- | --- | --- |
| Age | Continuous age at interview | Women who were engaged in commercial sex at 25 years or older were more likely to know serostatus and be on ART than those who started younger than 25 years^62^. HIV prevalence has been shown to increase with each year of age among women engaged in commercial sex^63^. Age at which sex work was initiated was not available, but age at interview was available. |
| Education | Highest level of education completed | Higher levels of education have been shown to be significantly associated with repeat testing among individuals with a previous HIV diagnosis.^64^ |
| Marital status | Marital status | In some settings, HIV prevalence and incidence rates are higher among single women compared to those who are married or living with their partner[59] |
| History of pregnancy | Continuous number of pregnancies | Women who attend antenatal appointments are more likely to receive HIV test and thus know their serostatus, receive counseling, start ART, and have VLS[60]. |
| Residence | Urban or rural | It may be more difficult for women engaged in commercial sex and living in rural locations to access and adhere to care[59]. |
| Food insecurity | Participant or household member went to sleep hungry in the last 4 weeks | Used as a proxy for socioeconomic status or wealth. Because it was a pooled multi-country analysis, each country’s wealth score and index are not comparable. However, factors like cost of testing are shown to be barriers to HIV testing uptake and delivery and ART uptake and delivery among PLHIV^64^. |
| Age at first sex | Continuous age at first sex | Younger age of first selling sex is shown to be associated with HIV prevalence^65^. |
| Number of sexual partners | Continuous number of sexual partners in the last 12 months | Used as a proxy for the number of clients and risk behaviors. Commercial sex client number is significantly associated with HIV infection^66^. |
| HIV testing history | Ever tested for HIV prior to the interview | If reported never testing for HIV prior to the interview, individuals were considered not engaged in the cascade. |
| Decision/empowerment norms | Person who usually makes decisions about health care; Person who generally decides how money received is spent | Decision making about health care and money were used as a proxy for empowerment. A woman who can make her own decisions about health care and how money is spent may be more likely to be engaged in the HIV care cascade. |
| Condom use | Condom used last time had sex for money and/or gifts | Inconsistent condom use among women engaged in commercial sex is considered a risk for HIV^68^. We hypothesize that women engaged in commercial sex who consistently use condoms are more likely to be engaged in the cascade. |

PHIA, Population-based HIV Impact Assessment surveys; WSS, women engaged in commercial sex; HIV, Human Immunodeficiency Virus; ART, antiretroviral treatment; VLS, viral load suppression; PLHIV, people living with HIV; TB, tuberculosis
